# Factors affecting rehabilitation of infants with Central Coordination Disorders during a three-month-long observation

**DOI:** 10.1186/s12887-021-03066-4

**Published:** 2021-12-18

**Authors:** Malgorzata Andrzejewska, Katarzyna Hap, Karolina Biernat, Edyta Sutkowska, Iwona Demczyszak, Dominik Marciniak, Natalia Kuciel

**Affiliations:** 1 Non-public Specialist Healthcare Institution Medi-Reh, Kalisz, Poland; 2 Department and Division of Medical Rehabilitation, Wroclaw Medical University, Borowska Street 213, 50-556 Wroclaw, Poland; 3 Department of Drugs Form Technology, Wroclaw Medical University, Wroclaw, Poland

**Keywords:** central coordination disorders, factors, rehabilitation

## Abstract

**Background:**

Central coordination disorders (CCD) encompass various abnormalities observed in infants but early therapy may have an impact on their condition. The aim was to seek factors that may affect the early results of therapy of infants with CCD.

**Methods:**

We analyzed the outcomes of a three-month period of rehabilitation of infants living with CCD. Children were treated at Non-public Specialist Healthcare Institution Medi-Reh in Kalisz in the period from 1 Jan 2014 to 31 Nov 2019. In our retrospective study results of three-month therapy of infants, aged 1 to 6 months, with CCD were analysed regards to the effectiveness and the potential impact of different factors. Therapy and assessment of children were conducted with the use of the Vojta method, which was performed during the first visit (WW) and the follow-up visit (after 3 months- 1WK). The analysis of the influence of various factors on the effect of therapy included: mother's age at the time of delivery, duration of breastfeeding, child APGAR, gestational age in which the child was born, sex of the child, birth weight, age of the child at WW, type of delivery, craniosacral therapy as an additive treatment.

**Results:**

Based on the examination results from 66 medical records it was demonstrated that after active period of the therapy, improvement was observed in 54 (81.81%) (p=0.48) children (condition during WW versus 1WK among the group). The sole factor impacting improvement after 3 months was the age of the child at WW, when the child started therapy. This factor significantly (p=0.002) increased the chance of achieving improvement - by 3.2 times (OR= 3,2; CI= 95). No statistically significant differences were shown for the other studied factors.

**Conclusions:**

Prompt implementation of rehabilitation in children with CCD provides a better chance of improving their motor function. The rehabilitation should be started as soon as possible after the diagnosis is constituted.

## Background

Central coordination disorders (CCD) are a broad term that describes abnormal motor development in children. As CCD is not a specific disease entity, and has only diagnostic and classification value, there are no epidemiological data that could be analysed in the context of its incidence. Evaluation of motor development at an early stage of life should be conducted on a regular basis to diagnose any possible abnormalities as soon as possible and begin rehabilitation [1].

The aim of paediatric interventions is to promptly provide care to a child that has been diagnosed with a medical problem requiring rehabilitation, often as early as from the time of its birth, for example in the case of patients with an increased risk related to pregnancy and birth.

In the case of disorders related to immature or damaged nervous system, we apply various methods, jointly referred to as the neurodevelopemental methods [2].

The method developed by prof. Vojta, which is classified as one of early neurophysiological treatments, constitutes a universal method applied in the diagnostic evaluation and rehabilitation of children with movement disorders. It comprises both diagnostic tests that help track the progress made by the child and therapeutic interventions [3]. This diagnostic and therapeutic concept includes three elements: observation of spontaneous movement, postural reactions and examination of primitive reflexes. Observation of spontaneous movement enables a thorough evaluation of the patient with regard to their sensorimotor function and developed skills. It all provides information about the patterns that are qualitatively and quantitatively disturbed, which subsequently makes it possible to determine the goals and methods of the therapeutic approach. Prof. Vojta also put in order and described responses to 7 postural reactions within particular age brackets in the first year of life [4]. These include: traction reaction, Landau reaction, axillary langer reaction, horizontal Collis reaction, Peiper-Isbert reaction, vertical Collis reaction Vojta reaction. Apart from that, diagnostic evaluation conducted on the basis of the Vojta method includes also: the Babkin, Galant and Rossolino reflex, the rooting reflex, the palmar and plantar grasp reflex, the doll's eye reflex, lower limb support, the automatic gait reflex [4, 5].

Another therapy used in rehabilitation of children is craniosacral method originating in osteopathy. In the simplest terms, the method consists in the physical therapist delicately touching specific areas of the infant's body with their hands [6].

This way, the therapist can feel abnormal tension in the tissue structures and mobilize the connective tissue surrounding the organs. The applied touch calms, soothes, relieves tension and gently stimulates the nervous system [7].

Both of these methods are classified as stimulus therapy and are used in rehabilitation of children with CCD. They can mitigate as well as fix the results of their abnormal development resulting from, for example, the labour. Nevertheless, even in children with a similar severity of disorders the response to treatment may differ in terms of time and effect [8].

Vojta therapy is widely used in physiotherapy for the treatment of various pathologies. Scientific evidence indicates positive effects of the Vojta therapy on the treatment of subluxation of the hip joint [9]. The benefits of Vojta therapy were demonstrated in patients with Sotos syndrome with developmental delay, where rehabilitation improved the quality of motor patterns and cognitive activation [10]. The effectiveness of the Vojta therapy was demonstrated in children with spastic cerebral palsy- the therapy improved the sitting position and diaphragm movement during inspiration [11]. Recent studies demonstrate the effectiveness of the Vojta method in patients with periventricular leukomalacia with delayed neuromotor development [12]. This article confirms the importance of physiotherapeutic interventions in infants with CCD. Physiotherapy is very well accepted as an important and integral part of the interdisciplinary treatment of, for example, children with meningo-spinal hernia [13] or autism spectrum disorders [14] but there are no recommendations as to which physiotherapy strategies should be used at different ages. There is a need to fill this information gap that will help neurologists and physiotherapists in the proper treatment of these groups of patients.

It is emphasized that an advanced age of the mother may increase the risk of congenital abnormalities and affect the relationship between the mother and the child, which may strengthen or weaken the reception of the external stimuli applied during the treatment by the child [15]. Breastfeeding duration also contributes to the movement development of children [16]. The week of gestation in which the child is delivered (preterm birth (preterm infant) is associated with underdevelopment of the CNS) [17], the APGAR score (the lower the score, the greater the risk of CCD) [18], the sex of the child (the risk of neurological dysfunctions is higher in boys) [19], birth weight (both underweight and macrosomia may have an impact on the development of CCD) [20] as well as the age of the child at the beginning of the treatment [21] are other variables that are considered in the context of their impact on the results of the treatment. Due to the increasing number of deliveries by Caesarean section, the impact of the type of delivery on the outcomes of rehabilitation is also taken into account. Researchers suggest that they may be affected by such factors as the anaesthetics used during the Caesarean section [22], which is, after all, a surgical procedure. The delay in enabling the skin-to-skin contact between the mother and the newborn, which may occur during a non-physiological delivery, may translate into the motor skills developed by the child during the therapy [23, 24].

Knowledge of such factors and the strength of their impact can be helpful in planning the therapeutic process in all cases involving modifiable factors.

## Aim of the paper

The aim of the paper was to seek factors that may affect early results of rehabilitation of infants (three-month observation) and assess the degree of their impact.

## Material and Methods

### Study design and setting

We analyzed the outcomes of a three-month period of rehabilitation of infants suffering from CCD, aged 1-6 months, which met the below-listed inclusion criteria, and were treated at NSZOZ MEDI-REH in Kalisz. The analysis involved archival materials (a retrospective study) from the medical histories of patients - children rehabilitated by one person - the leader of the study group - in the period from 1 January 2014 to 31 November 2019. The additional treatment, namely craniosacral therapy (if included), was also provided by one person - a certified specialist. Thanks to that it was possible to reduce the impact of the interfering factor, namely the skills of the therapist (the so-called human factor). Approval for the study was obtained from the Bioethics Committee of the Medical University of Wroclaw (consent number KB-108/2019).

The following inclusion criteria were applied:

- a score of 8 - 10 on the APGAR scale in the first minute of life, and no significant deterioration in the health of the child, significantly impairing the function of the neonate (e.g. sudden need for cardiac-pulmonary resuscitation), in the first 10 minutes of its life; changes other than decreasing the score obtained by the child by 1 point in a given time, if the neonate obtained 10 or 9 points in the APGAR scale, were considered to be a significant deterioration of its condition.
- no major birth defects that would significantly impair the development of the child and/or would require conducting genetic diagnosis.
- eligibility for rehabilitation confirmed by a medical rehabilitation physician at the centre;
- the age of the child at the time of the assessment of its eligibility for rehabilitation: 1-6 months (of life), calculated based on completed months of life.
- abnormal result of the Vojta test on admission to the centre, defined as at least 6 abnormal responses (marked as abnormal (AN), or delayed (OP) in the test report), with abnormal muscle tension, which indicates moderate to severe CCD;
- parental consent to the use of the medical history for the purposes of the project;
- available information from the first medical examination (during the first visit (WW)), and follow-up visit (1WK) on the determined date, maintaining the defined timeframe.
- Exclusion criteria (medical history was excluded from the analysis if even one of the following was met):
- children who obtained a score of < 8 points on the APGAR scale in the first minute of life as well as those who obtained 8 - 10 points in the APGAR scale, but their condition deteriorated significantly in the first 10 minutes of life, considerably impairing their function (e.g. sudden need for cardiac and pulmonary resuscitation) and causing a decrease in the APGAR score by more than 1 point, compared to the starting point.
- diagnosis of major (significant) birth defects, information on a major birth defect, congenital defect syndrome (e.g. Down Syndrome, Sotos Syndrome) and/or indications for consultation at a Genetics Clinic,
- opinion of the physician indicating that there is no need to rehabilitate the child using the Vojta method despite attending the consultation, or selection of other rehabilitation method by the physician;
- age < 1 month or > 6 months ;
- < 6 abnormal responses during the Vojta test at first eligibility visit, which indicates mild or very mild CCD;
- lack of parental consent to the use of documentation for the purposes of the study

### Participants

66 children who met the inclusion criteria were qualified for rehabilitation at the WW visit and completed the 3-month procedure. The rehabilitation and evaluation of children was conducted on the basis of the standard protocol adopted by Non-public Specialist Healthcare Institution Medi-Reh in Kalisz for diagnostic evaluation and treatment. According to the plan, the neurokinesiological examination using the Vojta method is performed at the centre during the first eligibility visit (WW) and during the follow-up visits, the first of which takes place after 3 months (1WK), and each next - after every 3 months following the previous visit (2WK, 3WK etc.).

### Variables

For the purposes of basic characterization and grouping of factors potentially affecting the results of rehabilitation, the so-called "maternal factors" and "child factors" were distinguished in the first place.

The following maternal factors were analysed:

- mother's age at the time of delivery
- duration of breastfeeding, from the moment of birth until the completion of the therapy

provided in completed weeks.

The following were analysed among child-related factors:

- the APGAR score obtained in the first minute of the child's life
- the week of gestation (gestational age) in which the child was born (Hbd)
- the sex of the child
- birth weight (BW)
- the age of the child at first eligibility visit, which was equivalent to the beginning of rehabilitation - calculated based on the months completed since birth.

Subsequently, the authors took into the type of delivery (natural vs Caesarean section) as well as the rehabilitation method (the Vojta method only vs the Vojta method combined with craniosacral therapy).

### Statistical analysis

Statistical analysis was conducted using Statistica 12 and Excel. Descriptive statistics were presented in tables, using measures of location: the mean, the median, the standard deviation, the minimum and the maximum. Distribution of variables was analysed using the Shapiro-Wilk W test. The impact of independent variables, including those encoded using the zero-one system, on variables of dichotomous type was verified using the single-factor logistic regression model, in which the Odds Ratio (OR) plays an important role. A p-value of ≤ 0.05 was deemed to be statistically significant [25, 26].

## Outcomes

For the purposes of the study, the authors used the results from first eligibility visit and 1^st^ follow-up visit. During first eligibility visit, after establishing the diagnosis and assessing the child's eligibility for rehabilitation, the specialist determines the aim of the treatment and the rehabilitation method (e.g. the Vojta method or the Vojta method combined with craniosacral treatment, which also concerned children whose histories were analysed). Depending on the outcomes, the method can be modified during subsequent visits. If, compared to first eligibility visit, the number of abnormal responses at 1^st^ follow up visit is lower (the Vojta method), it indicates that there has been an improvement.

### Basic characteristics

The analysed characteristics attributed to the mother and the child are presented in Table 1.

**Table 1:** Baseline characteristics of the study group including maternal factors and child- related factors

Risk/Protective Factor	mean	SD	median	Min	Max
**MATERNAL FACTORS**					
mother's age at the time of delivery (years)	30,70	4,94	31	20	43
duration of breastfeeding, (weeks)	16,92	15,07	12,5	0	47
**CHILD-RELATED FACTORS**					
the APGAR score	9,61	0,65	10	8	10
the week of gestation (gestational age) in which the child was born (Hbd)	38,33	1,9	39	31	41
birth weight (gram)	3300,53	532,96	3335	1600	4280
the age of the child at 1st WW (weeks)	3,21	1,1	3	1	5

In the study group, 27 (40.91%) children were delivered naturally, and 39 (59.09%) were delivered by a Caesarean section. In 52 (78.79%) children, the Vojta therapy was the sole method of rehabilitation, while rehabilitation of 14 (21.21%) infants consisted in combining the Vojta method and the craniosacral treatment, based on the decision of the consulting physician.

### Association between characteristics and clinical improvement

After 3 months of rehabilitation, improvement was observed in 54 (81.81%) children. There was no difference in the obtained outcomes of rehabilitation between the percentage of children delivered naturally - 21 (38.89%) infants - and those delivered by c-section - 33 (61.11%) infants (p = 0.48).

In the group of children rehabilitated with the use of the Vojta method only, improvement was observed in 43 (82.69%) infants, while in the group of patients in whom the Vojta method was combined with craniosacral therapy improvement was observed in 11 (78.57%) patients.

The results of the statistical analysis showed no statistically significant effect of the applied therapy on the improvement of the health of the treated patients (chi2 = 0.13, OR = 0.77; p = 0.72).

Both mother and child-related factors as well as the type of delivery and the rehabilitation method were then analyzed with regard to their impact on the obtained outcomes.

The only independent factor affecting the observed improvement after three months of rehabilitation was the child's age at the time of the beginning of the therapy (p < 0.01). This factor increased the chance of improvement by 3.2 times.

Other factors were not shown to have an impact on the obtained improvement evaluated at 1^st^ follow-up visit. The impact of the evaluated independent factors is presented in Table 2.

**Table 2:** The impact of the evaluated independent factors

Factor	*p*	Odd ratio	-95%CI	+95%CI
Mode of delivery	0,48	0,64	0,18	2,29
Sex of the child	0,53	1,51	0,41	5,48
Mother’s age at delivery (years)	0,53	0,96	0,84	1,09
Duration of breastfeeding (weeks)	0,16	1,04	0,99	1,09
APGAR score	0,28	1,63	0,67	3,99
Gestational age	0,62	0,91	0,63	1,31
Birth weight (grams)	0,49	1,00	1,00	1,00
Age of a child at the 1^st^ visit	0,002	3,20	1,55	6,58
Vojta therapy + craniosacral therapy	0,80	0,84	0,22	3,28
Vojta therapy	0,80	1,19	0,30	4,63

*p*  statistical significance, *CI *confidence interval

## Discussion

We have shown that the earlier age when therapy is started the higher percentage of improvement can be reached among patients. After a three-month-long observation, during the first follow-up visit and based on the assessment of the effects of rehabilitation, it was observed that the faster the treatment is introduced, the better it is for the child (chances of improvement increased by 3.2 times). During the early stage of development, it is possible to inhibit many inherently progressive disorders that without intervention could become established, or even progress over time. By introducing therapy, we can have also an impact on the expansion of synaptic connections [27, 28]. What is more most of the brain structures undergo myelination during the first year of life [29], and according to many authors [27, 30–34], it is most optimal to introduce therapy in the first three months of life (no later than in the sixth month of life). Such prompt intervention enables beneficial modification of the above-described processes, and even reversal of lesions within the CNS that have not yet become established [35]. Of course, therapy can also be introduced after that time [8, 36], but its effectiveness and the chance of complete reversal of pathologies decreases. It should be emphasized that children rehabilitated at the centre from which the analysed cases were obtained were referred for rehabilitation immediately after the diagnosis of abnormalities, and the mean age at which their rehabilitation began was a little over 3 months and did not exceed 5 months. This explains the very high percentage of improvements achieved after only three months.

According to many researchers, breastfeeding duration may play a significant role with regard to better rehabilitation results [35–44] as it impacts the formation of normal bacterial flora in the child's gastrointestinal tract [45–53]. The intestinal bacterial flora "communicates" with the central nervous system through nervous, immune and hormonal pathways [25, 26]. The impact of the intestinal microbiome on the brain is referred to as the gut-brain axis, and treatment strategies for some CNS disorders consisting in intestinal microbiome modification are currently in development. Researchers [26, 48, 54–60] also suggest that breastfeeding may be associated with the functioning of the child's nervous system due to the special interaction between the mother and the child that strengthens their bond and plays an important role in the child's emotional development, which also has an impact the rehabilitation process.

Even though c-sections should be performed only when medically necessary, the percentage of such deliveries is constantly increasing in many countries [61]. This procedure may affect not only the mother, but also the child, and its consequences are not necessarily immediately visible in infants. Some authors indicate that children delivered by Caesarean section may be more likely to ignore visual stimulation and may be more sensitive than those delivered naturally [62]. It is also suggested that Caesarean sections may have a negative impact on the occurrence and duration of involuntary reflexes [63]. In addition, Caesarean section, delays the "skin-to-skin" contact and may finally affect the psychomotor development of the child [24]. Procedures typical of all surgeries may also affect the development of the child (the impact of the drugs used during the labour), who has more difficulties in emotion management and shows an increased response to stress [24]. Since some authors emphasized that the method of delivery may have an impact on the rehabilitation outcomes [27, 64–68], we decided to determine whether such a relationship exists. In our short, three-month observation we did not demonstrate that the method of delivery had an impact on the outcome of rehabilitation. This is in line with another study, involving a twice as large group of children treated for congenital torticollis, the authors of which also did not demonstrate a relationship between the method of delivery and the outcomes of therapy. Within the framework of the same study, however, the said authors confirmed that the age at which diagnosis is made and rehabilitation is introduced has a significant impact on the results of treatment [69]. This observation is in line with our results.

Literature indicates that the outcome of rehabilitation can be affected by a number of variables. According to researchers, it is significantly affected by such factors as: the week of pregnancy in which the child was delivered, birth weight or the condition of the child after its birth (the score on the APGAR scale) [27, 30, 31, 37, 38, 66, 70–73]. It is particularly emphasized in the case of prematurely born children, in whom it was proven that the relationship between birth weight and the APGAR score was a significant factor determining psychomotor development [68]. These factors were taken into account during the analysis of the results of this study, but were not shown to be related to the outcomes of rehabilitation. It should be emphasized, however, that the APGAR score of 8 - 10, which constitutes an inclusion criterion in our study, is responsible for the so-called "good condition of a child". Therefore, it may have diminished the role of this scale in the obtained results of rehabilitation. For this reason, it seems advisable that subsequent prospective clinical studies involve larger groups of patients, divided into groups on the basis of, among others, this variable. Based on the retrospective material, the authors of the paper decided that only results of children who obtained a normal score on the APGAR scale after birth would be included in the analysis as this group is larger in size. In order to obtain groups of patients with lower APGAR scores that would be adequate in size, we would have to abandon the concept of analysing patients rehabilitated by one specialist only. As a result, the analysis could be affected by the above-described, unmeasurable human factor. The large diversity of such variables as the week of gestation in which the child was born, or its birth weight was also not shown to affect the effects of rehabilitation in our study. On the one hand, it may stem from the small number of children with extreme results (11 children who were born before the 38th week of pregnancy and 9 children with extreme, i.e. too high and too low, body weight), on the other - from the "mitigating" impact of a high APGAR score obtained by these children despite earlier delivery and low birth weight. Normal distribution shown for these parameters indicates that the analyzed group was representative for the clinical population.

Both therapies applied at the Centre (i.e. the Vojta method and the craniosacral method) are classified as stimulus therapies, meaning that external stimulation, in this case applied by a physical therapist, stimulates the nervous system of the child. Therefore, external factors may both strengthen and interfere with the reception of such stimulation and finally affect the outcome of the treatment. It seems that the skills of the therapist as well as their commitment and good contact with parents [74] who must perform the recommended activities at home after receiving a special training, have the greatest impact. It is very difficult to assess the skills, commitment and the influence of the therapist on parents as they are associated with a number of subjective factors. Therefore, the authors decided to exclude this biasing factor and analyse only the effects of rehabilitation of infants conducted by one person. The craniosacral therapy was also conducted by only one person. This way, the other factors could be analysed without any potential interferences.

The earlier rehabilitation is started, the better the therapy results. There is a need to assess if the same variable impact rehab effect if the APGAR score is lower. It seems that other studies, focusing on the impact of different variables described in this paper in groups of children with lower APGAR scores are recommendable as such patients may require much more radical action than children with normal scores on the scale.

## Conclusions

Prompt implementation of rehabilitation in children with CCD provides a better chance of improving their motor function. The rehabilitation should be started as soon as possible after the diagnosis is constituted.

### Acknowledge

We would like to thank the director of Non-public Specialist Healthcare Institution Medi-Reh in Kalisz, Poland, who gave her permission to conduct the study based on the Center records.

## Data Availability

The datasets used and/or analysed during the current study are available from the Andrzejewska Malgorzata on reasonable request.

## Abbreviations

CCD: Central Coordination Disorders
CNS: Central Nervous System
BW: birth weight
WW: first eligibility visit
WK: follow-up visit

## References

[1] Sidor-Piekarska B (2010). Early Intervention as Aiding the Development of a Child with Developmental Problems and Fiving Support to the Child's Parents. Roczniki Pedagogiczne.

[2] Karch D, Heinemann K, Physiotherapeutic interventions: Bobath, Vojta, and motor learning approaches. In: Christos P. Panteliadis (ed.), Cerebral palsy. A multidisciplinary approach, 3th edition, 2018; 16: 155-164.

[3] Bauer H, Appaji G, Mundt D (1992). *VOJTA neurophysiologic therapy*. Indian J Pediatr.

[4] Dimitros I (2004). Zafeiriou, Primitive reflexes and postural reactions in the neurodevelopmental examination. Pediatric Neurology.

[5] Orth H, Surowińska J (ed.), Terapia metodą Vojty, 1st edition, Wroclaw, Edra Urban&Partner, 2013.

[6] Downey PA, Barbano T, Kapur-Wadhwa R (2006). Craniosacral Therapy: The Effects of Cranial Manipulation on Intracranial Pressure and Cranial Bone Movement. Journal of Orthopaedic & Sports Physical Therapy.

[7] Raith W, Marschik PB, Sommer C (2016). General Movements in preterm infants undergoing craniosacral therapy: a randomised controlled pilot-trial. BMC Complementary and Alternative Medicine.

[8] Czenczek- Lewandowska E, Przygoda L, Szklarska-Witek I (2016). Changes in motor development in infants participating in rehabilitation based on Vojta method. Medical Review.

[9] Dwornik M, Kiebzak W, Zurawski A (2016). Vojta method in the treatment of developmental hip dysplasia—A case report. Ther. Clin. Risk Manag..

[10] Andrzejewska M, Sutkowska E, Kuciel N (2018). The rehabilitation of a child with a sotos syndrome. case report. Wiad. Lek..

[11] Ha S-Y, Sung Y-H (2018). Effects of Vojta approach on diaphragm movement in children with spastic cerebral palsy. J. Exerc. Rehabil..

[12] De-La-Barrera-Aranda E, Gonzalez-Gerez JJ, Saavedra-Hernandez M, Fernandez-Bueno L, Rodriguez-Blanco C, Bernal-Utrera C. Vojta Therapy in Neuromotor Development of Pediatrics Patients with Periventricular Leukomalacia: Case Series. Medicina. 2021;57:1149. 10.3390/medicina57111149 10.3390/medicina57111149 PMC8624747 34833367

[13] Aizawa CY, Morales MP, Lundberg C, Moura MC, Pinto FC, Voos MC, Hasue RH (2017). Conventional physical therapy and physical therapy based on reflex stimulation showed similar results in children with myelomeningocele. ArqNeuropsiquiatr.

[14] Grzadzinski R, Amso D, Landa R, et al. Pre-symptomatic intervention for autism spectrum disorder (ASD): defining a research agenda. J Neurodevelop Disord. 2021;13:49. 10.1186/s11689-021-09393-y 10.1186/s11689-021-09393-y PMC8520312 34654371

[15] Greg J, Duncan GJ, Kenneth THL, Rosales-Rueda M, Kalil A. Maternal Age and Child Development. Demography. 2018;55(6):2229–55. 10.1007/s13524-018-0730-3 PMC6392079 30387046

[16] Oddy W, Robinson M, Kendall G (2011). Breastfeeding and early child development: a prospective cohort study. Acta Peadiatr..

[17] You J, Shamsi BH, Hao M (2019). A study on the neurodevelopment outcomes of late preterm infants. BMC Neurology.

[18] Ehrenstein V (2009). Association of Apgar scores with death and neurologic disability. Clin Epidemiol..

[19] Dan B, Sex differences in neurodevelopmental disorders, Developmental Medicine& Child Neurology, 2021; 63(5): 492-292. 10.1111/dmcn.14853 33792030

[20] Yamada T, Akaishi R, Yamada T (2014). Risk of cerebral palsy associated with neonatal encephalopathy in macrosomic neonates. J Obstet Gynaecol Res..

[21] Zhang M, Gazimbi M, Chen Z (2020). Association between birth weight and neurodevelopment at age 1–6 months: results from the Wuhan Healthy Baby Cohort. BMJ Open.

[22] Boutsikou T (2011). A Malamitsi-Puchner A. Caesarean section: impact on mother and child, Acta Paedriatr..

[23] Gouchon S, Gregori D, Picotto A (2010). Skin-to-Skin Contact After Cesarean Delivery; an Experimental Study. Nurs Res..

[24] Pilch D (2015). The influence of birth modus on the emotional state of the mother, bonding and the newborn’s neurobehavioural state. J Life Sci.

[25] Kuciel N, Mazurek J, Czosnykowska-Łukacka M (2018). Stem cells in breast milk. Pediatr Pol.

[26] Edwards SC, Jędrychowski W, Butscher M (2010). Prenatal exposure to airborne polycyclic aromatic hydrocarbons and children's intelligence at 5 years of age in a prospective cohort study in Poland. Environ Health Perspect..

[27] Banaszek G (2004). Rozwój niemowląt i jego zaburzenia a rehabilitacja metodą Vojty.

[28] Makara-Studzińska M, Grzywa A, Ślipa B (2012). Plastyczność mózgu. Pol Merk Lek..

[29] Kułakowska Z, Konera W (2003). Wczesne uszkodzenie dojrzewającego mózgu.

[30] Banaszak G (2010). Metoda Vojty jako wczesna diagnoza i koncepcja terapii neurorozwojowej. Przegl Lek.

[31] Banaszek G (2005). Metoda Vojty jako wczesny skrining rozwoju niemowląt. Essentia Medica.

[32] Vojta V, Peters A (2006). Metoda Vojty.

[33] Surowińska J., Metoda Vojty. Praktyczny poradnik dla rodziców,1st ed., Warszawa, PZWL Wydawnictwo Lekarskie, 2021.

[34] Kanda T, Pidcoc FS, Hayakawa K (2004). Motor outcome differences between two groups of children with spastic diplegia who received different intensities of earlyonset physiotherapy followed for 5 years. Brain Dev..

[35] Czenczek-Lewandowska E, Przygoda L, Szklarska-Witek I (2016). ADHChanges in motor development in infants participating in rehabilitation based on Vojta method. Medical Review.

[36] Balewska-Juras K, Cywińska–Wasilewska G. Ocena wyników neurokinezjologicznego usprawniania metodą odruchowej lokomocji według Vojty, dzieci z zaburzeniami centralnej koordynacji nerwowej. Fizjoterapia Polska 2015;15(4):32-41.

[37] Dytrych G (2009). Analysis of motor development of premature born children with low body weight rehabilitated with the Vojta method. Neurologia Dziecięca.

[38] Meholjić-Fetahović A (2005). Importance of early rehabilitation using the Vojta method in symptomatic high risk infants. Med Arh..

[39] Pyta-Dulewicz A (2015). Wpływ pierwszej fazy odruchowego obrotu według Vojty na zakres ruchomości odcinka szyjnego u niemowląt. Ann Acad Med Siles..

[40] Dytrych G (2015). Wpływ stymulacji metodą Vojty na rozwój psychoruchowy dzieci z zespołem Downa. Neurologia Dziecięca.

[41] Jung W, Landenberger M, Jung T, Ti et al.;. Vojta therapy and neurodevelopmental treatment in children with infantile postural asymmetry: a randomized controlled trial. J PhysTherSci. 2017;29(2):301-306. 10.1589/jpts.29.301 PMC5332993 28265162

[42] Sadowska L, Szpich E, Wójtowicz D (2006). Odpowiedzialność rodzicielska w procesie rozwoju dziecka niepełnosprawnego. Przegląd Medyczny Uniwersytetu Rzeszowskiego, Rzeszów.

[43] Smigiel R, Kaczan T (2012). Wczesna interwencja i wspomaganie rozwoju u dzieci z chorobami genetycznymi.

[44] Wojtowicz D, Dolyk B (2006). Effects of Vojta therapy on the hip joints' mobility in infants with a central coordination disturbance. Fizjoterapia.

[45] Andreas N, Kampmann B, Mehring L-DK (2015). Human Breast Milk: A Review on Its Composition and Bioactivity. Early Hum Dev..

[46] Bardanzellu F, Peroni D, Vassilios FV (2020). Human Breast Milk: Bioactive Components. From Stem Cells to Health Outcomes..

[47] Witkowska-Zimny M, Kamińska-El-Hassan E (2017). Cells of Human Breast Milk. Cell Mol Biol Lett..

[48] Belfort M (2017). Science of Breastfeeding and Brain Development. Breastfeed Med..

[49] Underwood M, Mukhopadhyay S, Lakshminrusimha S, Bevins C (2020). Neonatal intestinal dysbiosis. Journal of Perinatology.

[50] Cesar G, Victora R, Barros A (2016). Breastfeeding in the 21st century: epidemiology, mechanisms, and lifelong effect. Lancet Breastfeeding Series Group, Lancet..

[51] Janczewska I, Domżalska-Popadiuk I (2014). Znaczenie kolonizacji bakteryjnej przewodu pokarmowego noworodków donoszonych urodzonych drogą cięcia cesarskiego. Ann Acad Gedan..

[52] Jakobsson H, Abrahamsson T, Jenmalm M (2014). Decreaesed Gut micro biota diversity, delayed Bacteroidetes colonisation and reduced Th1 responses in infants delivered by caesarean section. Gut.

[53] Gizzo S, Gangi D, Saccardi C (2012). Epidural Analgesia During Labor: Impact on Delivery Outcome, Neonatal Well-Beimg, and Early Breastfeeding. Breastfeed Med..

[54] Lessing-Pernak J (2010). Znaczenie przebiegu porodu i wczesnego kontaktu matki z dzieckiem dla rozwoju przywiązania. Perinatologia, Neonatologia i Ginekologia.

[55] Pilch D (2015). Wpływ modułu porodowego na stan emocjonalny matki, tworzenie więzi z dzieckiem i stan neurobehawioralny noworodka. Pom J Life Sci..

[56] Sandall J, Tribe R, Avery L (2018). Short-term and long-term effects of caesarean section on the health of women and children. Lancet.

[57] Moore E, Bergman N, Anderson G, et al. Early skin-to-skin contact for mothers and their healthy newborn infants. Cochrane Systematic Review-Intervention. 2016. 10.1002/14651858.CD003519.pub4 PMC6464366 27885658

[58] Karimi F, Miri H, Khadivzadech T (2020). The effect of mother-infant skin-to-skin contact immediately after birth on exclusive breastfeeding: a systematic review and meta-analysis. J Turk Ger Gynecol Assoc..

[59] Sundin C, Mazac L (2015). Implementing Skin-to-Skin Care in the Operating Room After Cesarean Birth. MCN Am J Matern Child Nurs..

[60] Mockun J, Olszewska J (2017). Wpływ cięcia cesarskiego na rozwój psychomotoryczny dziecka. Pielęgniarstwo Polskie.

[61] Boerma T, Ronsmans C, Dessalegn Y (2018). Global epidemiology of use of and disparities in caesarean sections. Lancet.

[62] Grivell R (2011). Dodd J. Short- and long-term outcomes after cesarean section, Expert Rev Gynecol.

[63] Bartett D, Piper M, Okun N (1997). Primitive reflexes and the determination of fetal presentation at birth. Early Hum Dev..

[64] Bagnowska K (2014). Factors affecting the efficacy of rehabilitation NDT-Bobath children born prematurely. Nowa Pediatria.

[65] Minguez-Milio J, Alcazar J, Auba M (2011). Perinatal outcome and long-term Follow-up of extremely low birth weight infants depending on the mode of delivery. J Matern Fetal Neonatal Med..

[66] Sadowska L, Gomulska K, Krefft A (2005). Wczesna, syntetyczna diagnostyka mózgowego porażenia u dzieci ryzyka leczonych metodami neurorozwojowymi. Fizjoterapia Polska.

[67] Jajor J (2012). Płeć a rozwój funkcjonalny dzieci w wieku do 2 lat. Nowiny Lekarskie.

[68] Sitarz L, Pop T, Opalińska I (2011). Ocena rozwoju psychomotorycznego niemowląt urodzonych przedwcześnie w pierwszym półroczu życia. Young Sport Science of Ukraine.

[69] Young Jung A, Young Kang E, Hoon Lee S (2015). Factors That Affect the Rehabilitation Duration in Patients whit Congenital Muscular Torticollis. Annals of Rehabilitation Medicine..

[70] Plagens-Rotman K, Bączyk K, Kubiak S (2011). Krwawienia wewnątrzczaszkowe u noworodków z ekstremalnie małą urodzeniową masą ciała. Nowiny Lekarskie.

[71] Wolosiewicz M, Kowalski I, Tomaszewski W (2005). Sprawność motoryczna pacjentów z mózgowym porażeniem dziecięcym i u dzieci zdrowych w zależności od pourodzeniowej oceny w skali Apgar. Fizjoterapia Polska.

[72] Cembrzyńska J, Jabłeka A, Niewiadomski P, i wsp. (2014). Ocena rozwoju psychomotorycznego i usprawnianie dziecka przedwcześnie urodzonego. Rehabilitacja.

[73] Meholjić-Fetahović A (2006). Prematurity as a motor development risk factor. Med. Arh.

[74] Dirks T, Hadders-Algra M (2011). The role of the family in intervention of infants at high risk of cerebral palsy: a systematic analysis. Dev L Med Child Neurol..

[75] Lubecki M (2011). The Polish model of rehabilitation accepted and recommended by WHO, Hygeia. Public Health.

